# S213. CAN PATIENTS WITH TREATMENT RESISTANT SCHIZOPHRENIA RELIABLY REPORT NEGATIVE SYMPTOMS? A PILOT STUDY USING THE SELF-EVALUATION OF NEGATIVE SYMPTOMS SCALE

**DOI:** 10.1093/schbul/sby018.1000

**Published:** 2018-04-01

**Authors:** Jean-Pierre Lindenmayer, Abraham Goldring, Amanda Hefner, Anzalee Khan, Amod Thanju

**Affiliations:** 1 New York University; 2 Manhattan Psychiatric Center

## Abstract

**Background:**

The Self-Evaluation of Negative Symptoms (SNS), a 20-item self-rating scale,was developed to assess the subjective experience of negative symptoms by schizophrenia patients. The reliability and validity of the translated French version of the SNS was examined in a sample of outpatients in an US site with schizophrenia and schizoaffective disorders (Dollfus et al., 2015). The author found that the SNS had good psychometric properties and demonstrated that the patients’ ratings were highly correlated with observer ratings, which contradicts the expected lack of reliability of patient reported symptoms in patients with schizophrenia. However, the patients included in the study were stable outpatients with high levels of functioning as compared to lower functioning patients. It remains to be explored whether patients with lower levels of functioning are equally able to identify their negative symptoms in a reliable fashion. The aim of the present study was to first evaluate the reliability of the novel tool of self-evaluation of Negative Symptoms (SNS) and to examine its correlation with observer ratings of negative symptoms in a sample of inpatients with ICD 10 schizophrenia or schizo-affective disorder who function at a low level of overall cognition. It was our goal to examine if chronic, low functioning patients are able to complete the instrument without assistance, providing clinically meaningful information with respect to their own perception of negative symptoms.

**Methods:**

Patients who met DSM-5 criteria for schizophrenia or schizoaffective disorder were included in the study. All patients will provide written informed consent. Patients were administered the SNS assessment at two time points, separated by 1 one week, followed by other concurrent evaluations: the 16-Item Negative Symptom Assessment (NSA-16), a validated clinical assessment for negative symptoms, the CGI-S, WRAT, BACS, and the CDSS. To examine the internal consistency of the SNS. Cronbach’s alpha was calculated for the 20 items and the 5 sub scores at both times. Correlation analyses were performed to examine the convergent validity of the SNS with the observer rated negative symptom scale. Convergent and discriminant validities were tested with Pearson’s correlations. The test-retest reliability of the SNS will be tested by intraclass correlation coefficients (ICCs).

**Results:**

Fourteen patients with schizophrenia or schizoaffective disorder according to DSM-5, and a mean age of 43.00 (12.48) were evaluated. 66.67% of subjects were African American. Cronbach’s coefficient of the SNS (α = 0.791) showed good internal consistency. The SNS did not show significant correlations with the NSA-16 (r = 0.207, p = 0.497), the NSA global score (r = 0.390, p = 0.296), nor the Clinician Global Impression on the severity of negative symptoms (r = -0.264, p = 0.383). SNS scores did not correlate with level of insight as measured by the SUMD (r = -0.51, p = 0.870), Motor Functioning deficits as measured by the SAS (r = 0.227, p = 0.456). The intrasubject reliability of the SNS revealed good intraclass correlation coefficients (ICC = 0.780). Test-Retest was significant at 0.791, p = 0.004, with a significant change at t(12) = 3.923, p = 0.002.

**Discussion:**

Our pilot study suggests that the agreement between self-rating and observer-rating of negative symptoms in patients with treatment resistant schizophrenia is rather low as. Patients also evaluated the severity of their negative symptoms rather differently. Reasons for this discrepancy will be discussed, in particular, in the context of low levels of illness insight as well as the psychometric qualities if the SNS.

